# A Systematic Literature Review on Distributed Machine Learning in Edge Computing

**DOI:** 10.3390/s22072665

**Published:** 2022-03-30

**Authors:** Carlos Poncinelli Filho, Elias Marques, Victor Chang, Leonardo dos Santos, Flavia Bernardini, Paulo F. Pires, Luiz Ochi, Flavia C. Delicato

**Affiliations:** 1 Institute of Computing, Universidade Federal Fluminense, Av. Gal. Milton Tavares de Souza, São Domingos, Niterói 24210-310, RJ, Brazil; carlosponcinelli@gmail.com (C.P.F.); eliaslawrence@id.uff.br (E.M.J.); lmrsantos@gmail.com (L.d.S.); fcbernardini@ic.uff.br (F.B.); paulo.pires@ic.uff.br (P.F.P.); satoru@ic.uff.br (L.O.); 2 Department of Operations and Information Management, Aston Business School, Aston University, Birmingham B4 7ET, UK; victorchang.research@gmail.com

**Keywords:** machine learning, artificial intelligence, distributed, edge intelligence, fog intelligence, Internet of Things

## Abstract

Distributed edge intelligence is a disruptive research area that enables the execution of machine learning and deep learning (ML/DL) algorithms close to where data are generated. Since edge devices are more limited and heterogeneous than typical cloud devices, many hindrances have to be overcome to fully extract the potential benefits of such an approach (such as data-in-motion analytics). In this paper, we investigate the challenges of running ML/DL on edge devices in a distributed way, paying special attention to how techniques are adapted or designed to execute on these restricted devices. The techniques under discussion pervade the processes of caching, training, inference, and offloading on edge devices. We also explore the benefits and drawbacks of these strategies.

## 1. Introduction

Nowadays, with the rise of the Internet of Things (IoT), a large number of smart applications are being built, taking advantage of connecting several types of devices to the internet. These applications will generate a massive amount of data that need to be processed promptly to generate valuable and actionable information. Edge intelligence (EI) refers to the ability to bring about the execution of machine learning tasks from the remote cloud closer to the IoT/Edge devices, either partially or entirely. Examples of edge devices are smartphones, access points, gateways, smart routers and switches, new generation base stations, and micro data centers.

Some edge devices have considerable computing capabilities (although always much smaller than cloud processing centers), but most are characterized by very limited capabilities. Currently, with the increasing development in the area of MEMS (Micro–Electro–Mechanical Systems) devices, there is a tendency to carry out part of the processing within the data producing devices themselves (sensors) [1,2,3,4]. There are certainly several challenges involved in performing processing on resource-limited devices, including the need to adapt complex algorithms and divide the processing among several nodes.

Therefore, in Edge Intelligence, it is essential to promote collaboration between devices to compensate for their lower computing capacity. Some synonyms of this concept found in the literature are: distributed learning, edge/fog learning, distributed intelligence, edge/fog intelligence and mobile intelligence [5,6,7].

The leverage of edge intelligence reduces some drawbacks of running ML tasks entirely in the cloud, such as:

- High latency [8]: offloading intelligence tasks to the edge enables achievement of faster inference, decreasing the inherent delay in data transmission through the network backbone;
- Security and privacy issues [9,10]: it is possible to train and infer on sensitive data fully at the edge, preventing their risky propagation throughout the network, where they are susceptible to attacks. Moreover, edge intelligence can derive non-sensitive information that could then be submitted to the cloud without further processing;
- The need for continuous internet connection: in locations where connectivity is poor or intermittent, the ML/DL could still be carried out;
- Bandwidth degradation: edge computing can perform part of processing tasks on raw data and transmit the produced data to the cloud (filtered/aggregated/pre-processed), thus saving network bandwidth. Transmitting large amounts of data to the cloud burdens the network and impacts the overall Quality of Service (QoS) [11];
- Power waste [12]: unnecessary raw data being transmitted through the internet demands power, decreasing energy efficiency on a large scale.

The steps for data processing in ML vary according to the specific technique in use, but generally occur in a well-defined life cycle, which can be represented by a workflow. Model building is at the heart of any ML technique, but the complete life cycle of a learning process involves a series of steps, from data acquisition and preparation to model deployment into a production environment. When adopting the Edge intelligence paradigm, it is necessary to carefully analyze which steps in the ML life cycle can be successfully executed at the edge of the network. Typical steps that have been investigated for execution at the edge are data collection, pre-processing, training and inference.

Considering the aforementioned steps in ML and the specific features of edge nodes, we can identify many challenges to be addressed in the edge intelligence paradigm, such as (i) running ML/DL on devices with limited resources, (ii) ensuring energy efficiency without compromising the inference accuracy; (iii) communication efficiency; (iv) ensuring data privacy and security in all steps; (v) handling failure in edge devices; and (vi) dealing with heterogeneity and low quality of data. In this paper, we present the results of a systematic literature review on current state-of-the-art techniques and strategies developed for distributed machine learning in edge computing. We applied a methodological process to compile a series of papers and discuss how they propose to deal with one or more of the aforementioned challenges.

The purpose of this survey is to present the outcome of a recent literature review on EI, identify key components, and analyze the retrieved studies thoroughly, correlating techniques, strategies, frameworks and application domains. To achieve this end, we first present the challenges we found on EI; secondly, we discuss all techniques and strategies we found related to pre-processing federated learning and scheduling; thirdly, we describe the frameworks we found for supporting EI, as well as which group of techniques and strategies they are mainly related to; and fourthly, the paper presents a taxonomy of application domain areas, which involves industry, surveillance, security, intelligent transport systems, and health and energy management. We also present a comparison of our work with related works (surveys and others) we found in the literature, as well as presenting open issues and future directions.

**Figure 1:**
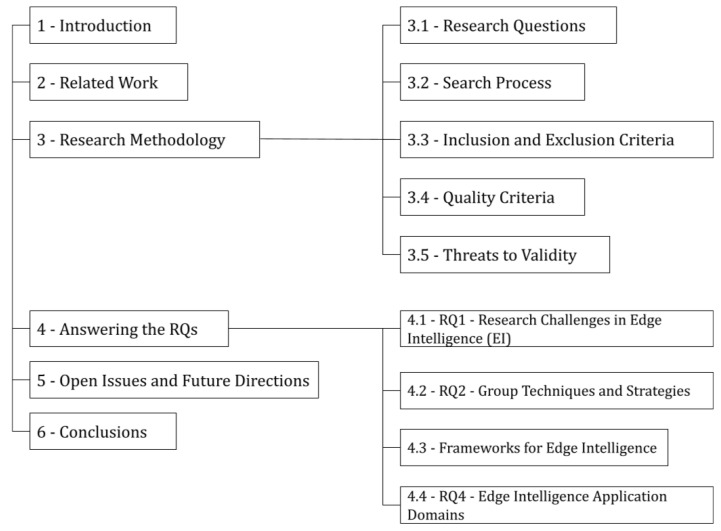
A schematic overview of the organization (structure) of this paper.

The rest of this paper is organized as follows: Section 2 describes related surveys we found in literature, as well as a comparison with this one. Section 3 approaches our research methodology to support the reproducibility of this systematic review. Section 4 presents our findings aligned to our Research Questions (RQs), Section 3. More specifically, regarding RQ2, we would like to draw attention to the nine groups of techniques and strategies we found (federated learning, model partitioning, right sizing, edge pre-processing, scheduling, cloud pre-training, edge only, model compression, and others), as well as the six application domainsw of edge intelligence we found (Industry, Surveillance, Security, Intelligent Transportation Systems, Health and Energy Management). Section 5 presents the open issues and future directions in EI. Finally, Section 6 presents the conclusions of this work. Figure 1 shows a schematic overview of our paper structure.

## 2. Related Work

Some surveys have been published that address the edge intelligence subject recently. However, they adopt different perspectives from the one adopted in this SLR. Al-Rakhami et al. [13] propose and analyze a framework based on the distributed edge/cloud paradigm using docker technology which provides a very lightweight and effective virtualization solution. This solution can be utilized to manage, deploy and distribute applications onto clusters (e.g., small board devices such as Raspberry PI). It is able to provide an advantageous combination of various benefits and lower costs of data processing performed at the edge instead of central servers. However, the authors base their proposal on experiments to support the proposal of a new framework. The research does not mention any of the nine groups of techniques we present in our work.

Wang et al. [14] survey is centered on the connection between Deep Learning and the edge, either to apply DL in optimizing the edge or to use the edge to run DL algorithms. The study is divided into five fronts: DL applications on edge; DL inference in edge; edge computing for DL; DL training at the edge; DL for optimizing the edge. The paper discusses hardware and virtualization aspects. Concerning the (groups of) techniques and strategies, it is more restricted to Federated Learning and the optimization of the edge with DL. In contrast, in our survey we discuss further aspects, focusing on edge pre-processing, training, inference and offloading. Furthermore, the authors did not explicitly group the techniques as we did.

Xu et al. [10] approach edge intelligence under the perspectives of edge caching, edge training, edge inference, and edge offloading in a very comprehensive way. We discuss all these aspects in our work but explore additional techniques, and strategies related to pre-processing, federated learning, and scheduling. One intersection of this paper with ours is the overlap of three groups of techniques we present (Federated Learning, Edge Pre-processing and Scheduling). However, we deepened our discussion into more groups of techniques.

The work presented by Zhou et al. [15] covers artificial intelligence to edge AI, showing a generalized representation of application architecture used in the lifecycle management of ML. In the edge layer: sensors/actuators; edge analytics; logging and monitoring. In the fog layer: visualization; live streaming engines; batch processing; data ingestion; storage and ML model development platforms and libraries. Our paper approaches several more domains in which edge intelligence is used, which are not present in this survey. Compared to these other surveys, we analyze the literature more comprehensively, including a discussion on application domains of edge intelligence and their correlation with identified techniques. This is also an excellent source of research in our scope, for dealing with applications of artificial intelligence applied to edge or edge intelligence. Although there is an intersection of this paper with ours in three groups of techniques we present (Federated Learning, Model Compression and Model Partitioning), they did not present the other six we discuss.

Verbraeken et al. [16] provide an extensive overview of the current state-of-the-art in terms of outlining the challenges and opportunities of distributed machine learning over conventional machine learning, discussing the techniques used for distributed machine learning. The paper follows the same line of research of Wang et al. [14], with a focus on machine learning applied to the distributed environment. To this end, it makes inroads into the various types of algorithms to solve problems using ML. However, the article does not refer to any of the nine groups of techniques that are described in our paper for edge applications. It can be considered a cornerstone article in the edge intelligence field to tackle questions of challenges, frameworks and application domains.

In this scenario, the contribution of the current manuscript to the existing surveys and review papers, when compared to the research published in 2020, is mainly in terms of the groups of techniques and strategies. Table 1 shows the comparison between our work and the other surveys mentioned in this section. In summary, the main gaps of the analyzed works are focused on aspects such as “Techniques and Strategies” on the edge. The table also shows the aspects of “Challenges”and “Different Application Domains”, where edge intelligence can be used.

To show the difference of our work compared to the related work we found, we used the six challenges, eight groups of techniques and strategies and six application domains that emerged from our SLR. Considering the six challenges we describe in this work, we observed that [13] does not even mention any challenge questions, [14,16] only address one of the challenges we describe, [10] addresses all the challenges we also discuss and [15] addresses two of these challenges. On the other hand, when considering the groups of techniques, [10] describes only three of the eight groups we present and [13] presents two of them, [14,15], four of them, and [16] does not cite any of them. Finally, considering the six application domains we discuss, [10,15,16] do not discuss any of them, [13] discusses only one and [14] discusses four of them. Table 1 shows these numbers. In this way, our SLR is more complete than all these related works.

Regarding the framework, our paper presents several frameworks that were developed to implement techniques and strategies from different groups that we identified and as a source for other EI works. Our work does not propose any framework. We just organize and compile information from a considerable numbers of frameworks from the researched literature that has contributed to this field of research. We intend to elaborate an aggregated base so that new works can use this research as a reference source. It is a survey paper, not a research paper, and our goal is to report the state of the art of the field.

## 3. Research Methodology

The research methodology used in this paper consists of a Systematic Literature Review (SLR), where a rigorous protocol of searching the literature is defined and applied to extract information that answers specific research questions. The use of this methodology enables impartial results and an auditable process. This section details the methodology used in the review.

According to Brereton et al. [17], an SLR is performed procedurally through distinct processes. This proposal includes an initial phase called ’Plan Review’, which includes: (i) specifying research questions; (ii) developing review protocol; (iii) validating review protocol. In the second phase, ’Conduct Review’, the following are carried out: (iv) identifying relevant research; (v) selecting primary studies; (vi) assessing study quality; (vii) extracting required data; (viii) synthesising data. In the last phase, ’Document Review’, the activities of producing and validating the reports with the reviewed findings are performed, respectively: (ix) writing of the review report, and (x) validating the report.

### 3.1. Research Questions

The definition of the research questions is the most important part of an SLR, since they guide all further steps of the review. The goal is to formulate questions that will be answered by the review of primary works retrieved from the literature.

To define the research questions, a thorough search was conducted to list all subjects that were being addressed in the area of Distributed Machine Learning in Edge Computing. Then, we identified research gaps that were not addressed by other surveys. The questions shown in Table 2 were formulated based on this list of subjects.

### 3.2. Search Process

After defining the research questions, we began the search for articles on the Scopus [18] online library of scientific papers, which enables advanced search according to predefined input criteria. The following string was used in the search for papers:

“edge intelligence” OR “edge artificial intelligence” OR “fog intelligence” OR “fog artificial intelligence"

In our research, we considered the use of Scopus as the main and unique source of research, taking into account some aspects: subject, size, type of record and citation search. According to Gusenbauer and Haddaway [19], who make a comparison between 28 academic consultation bases and evaluate 26 requisites, Scopus has a significant content depth, which motivated our choice of using Scopus as the source for our literature search.

We also chose Scopus due to over 10% of the Scopus database comprising conference papers (over 9.5 million), of which 2.5 million are published in journals, book series and other sources, 16 million author profiles, 70,000 affiliation profiles and 1.7 billion cited references dating back to 1970 [20]. The publication categories include books, journals, articles and conference papers in the field of computer, information science; medical and health sciences; physical sciences; mathematical sciences; economics; and seventeen others. The number of active titles indexed by Scopus vs. the nearest competitor based on geographical region is as follows: 54% in North America, 236% in the Middle East and Africa; 75% in Western Europe; 220% in Eastern Europe and Russia; 193% in Latin America; 265% in the Asia Pacific and 225% in Australia and New Zealand.

### 3.3. Inclusion and Exclusion Criteria

As the search returned a huge number of articles, some criteria for inclusion and exclusion of initial studies were necessary to select the ones that should be the focus of a more in-depth analysis. The inclusion and exclusion criteria used in this work are, respectively, presented in Table 3 and Table 4.

Regarding Table 4, a filter called relevance criteria was created to verify the relevance of any selected study, based on the number of citations. To apply the inclusion criteria, the retrieved papers were subject to peer review, where two researchers independently analyzed the same paper, which was accepted for inclusion only when both researchers agreed with its selection.

### 3.4. Quality Criteria

After applying the inclusion and exclusion criteria described in the previous subsection, an additional step involved filtering selected works according to a quality assessment, where the following criteria were proposed:

- QC1—Is there a clear definition of the research objectives?
- QC2—Is the proposed architecture/algorithm/protocol fully and properly explained?

Articles that did not meet these criteria were excluded.

### 3.5. Selection Process Steps

In this survey, the three main phases proposed by Brereton et al. [17] were followed: Plan Review, Conduct Review and Document Review. We conducted ten activities. In our Plan Review phase, the research questions were defined and the search string was tailored and applied. A total of 1560 scientific papers were retrieved. The results were inserted into a table to lay the groundwork for the subsequent steps. The Conduct Review phase should comprise the activities of identifying relevant research, selecting primary studies, assessing study quality, extracting required data and synthesizing data.

From those 1560 initial studies, 615 were selected to undergo the inclusion and exclusion criteria. This selection was based on the relevance of the papers regarding the number of citations. Thus, each researcher was responsible for evaluating a fair number of papers. This first analysis was based on the examination of the abstracts. The first round of analysis resulted in the exclusion of 291 papers. Then, the included articles were subject to the second round of analysis conducted by a different researcher, resulting in the elimination of 110 additional works. Twelve new papers were included by specialists as noteworthy.

**Figure 2:**
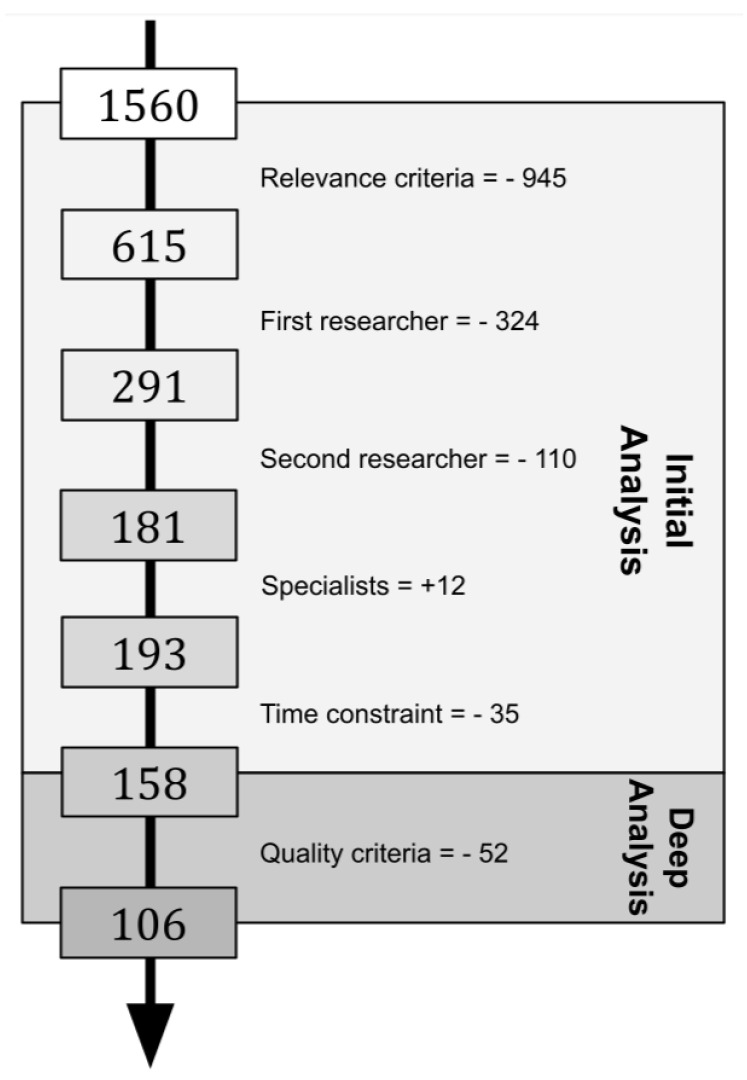
Number of papers excluded step by step in the SLR.

In the end, a total of 106 papers—given the constraint of time—was selected for a complete examination. Figure 2 summarizes the number of articles excluded/included in each step described above. Data regarding the selected papers were extracted to another table. The fields included in the data extraction table are listed below:

- Paper, Application Domain, Main Challenges, Technique and Strategies, Frameworks and Notes.

The third and final phase, Document Review, involves reporting the review findings, aiming at providing answers to the posed research questions. Considering the four defined RQs, Section 4.1 presents our findings regarding RQ1, Section 4.2 reports the findings related to RQ2, Section 4.3 provides answers to RQ3 and Section 4.4 discusses application domains, the target of RQ4.

### 3.6. Threats to Validity

The conducted systematic mapping and its results may have been affected by some threats to validity. In the following, we discuss some of these limitations.

The completeness of this systematic mapping may have been affected by missing relevant studies. To reduce this threat, we used electronic databases that are among the most relevant available sources in computer science and engineering. However, there are still limitations:

- Some studies may have been missed due to technical limitations of the automated search engines, an issue that is out of our control.
- The selected electronic databases do not represent an exhaustive list of publication sources, so other databases might also be included.
- We did not perform snowballing, a useful technique that consists of checking the reference lists of the read studies aiming to find additional studies that were not retrieved in the automated search procedure. Therefore, other possibly relevant studies could have been identified and considered in this systematic mapping.

## 4. Answering the RQs

In the following subsections, we present our findings related to each RQ described earlier.

### 4.1. RQ1—Research Challenges in Edge Intelligence (EI)

In this section, we summarize the challenges faced by the Edge Intelligence (EI) paradigm that the analyzed studies either mentioned or aimed to tackle. The discussion presented in this section aims to provide answers to RQ1: What are the main challenges and open issues in the distributed learning field?

As mentioned earlier, performing ML techniques at the edge of the network promises to bring several benefits, but it raises several challenges. As this field is still in its beginning, solutions to such challenges are still being investigated. The surveyed studies tackle several challenges, which can be broadly grouped into six categories, displayed in Table 5 and described in what follows.

CH1 consists of dealing with the typical low processing power of edge devices. Edge devices often have little processing capacity, mainly when compared to the powerful data centers at the cloud. On the other hand, many ML applications require high computational power that outweighs the possibilities of resource-constrained IoT and edge devices. Limited resources also include memory and storage capacities. NN and ML algorithms generally require storing of and access to a handful of parameters that describe the model architecture and weight values forming the classification model. With limited storage, it may not be possible to have continued access to the original training data, or the data may have been removed altogether to free up space. Therefore, a significant challenge is reducing memory access and storing the data locally to avoid costly reading and writing to external memory modules.

CH2 consists of ensuring the energy efficiency of edge devices without compromising the accuracy of the system. In general, the higher the complexity of the required processing, the more energy is consumed. Edge devices can be battery-powered. In these cases, the energy consumption of algorithms must be minimized to reach energy efficiency. However, this should be done with care so as not to compromise the quality of the data generated and the decisions/inferences made. So, there is an important trade-off to be managed.

CH3 concerns communication issues, where edge intelligence models must consider that the devices might face poor connectivity. In such cases, the model update time in training tasks may be delayed. Valerio, Passarella and Conti [21] claim that the inference is highly sensitive to the available bandwidth in communication. Challenges in communication include network traffic, fluctuations in the bandwidth, intermittent or unavailable connectivity.

CH4 is related to data privacy and security. Several applications in edge intelligence handle sensitive data, such as healthcare. Thus, distributed ML algorithms must be able to preserve user privacy and information security when data are transferred throughout the devices. Distributed Edge-Intelligence (EI) has multiple points of vulnerability to possible malicious attacks or leakage of confidential or important data in the ML workflow.

CH5 is the challenge posed by failures in edge devices. Since devices might fail at some point, the distributed algorithm must consider ways to overcome this situation. Lastly, heterogeneity and lack of quality in available data rise challenge CH6. For most ML algorithms, especially in supervised machine learning, high accuracy depends on the high quality of training data. However, this often does not apply in edge intelligence scenarios, where the collected data are sparse and unlabelled [10]. Distributed edge intelligence can handle data from different sources in different formats and is subject to noise. The application must handle noise and heterogeneity in the sensed data used as input to attain good accuracy.

Table 6 presents references to each of the described challenges, as well as studies that propose approaches to tackle these challenges. This table aims to only show an overview on the number of papers by each challenge. We can observe that challenge CH1 is the one with more papers present in literature. All of the cited works are better described later in this paper.

### 4.2. RQ2—Techniques and Strategies

The discussion presented in this section aims to provide answers to Research Question 2: What are the techniques and strategies currently used in distributed learning?

In our discussion, we focus on three main aspects, namely: (i) the system architecture, (ii) how the ML tasks are distributed among the devices, and (iii) the underlying adopted techniques. We classify the several approaches used in distributed learning based on these three aspects. We identified nine groups of techniques and strategies, described in what follows: Federated learning; Model partitioning; Right-sizing; Edge pre-processing; Scheduling; Cloud pre-training; Edge only; Model Compression; and Other techniques.

#### 4.2.1. Federated Learning

One of the most well-known and commonly implemented approaches in the EI research field is Federated Learning (FL). FL is based on the concept of Distributed Selective Stochastic Gradient Descent (DSSGD), introduced by Shokri [76] in 2015, and initially related to privacy-preserving in deep learning. This approach allows each part of the system to keep its local model private while iteratively updating it by integrating gradients of others through a parameter server. According to Lyu et al. [72], DSSGD exploits the fact that SGD can be parallelized and executed asynchronously.

As reported by McMahan et al. [77], the use of FL enables to train ML models on private client data through the iterative communications of parameters between the server and clients. The whole process begins with the initialization of a random global model in the server. Then, iteratively, the server sends the parameters to random clients, which must update the model with their own data and upload the new values to the server that averages the updated ones and replaces the global model with the averaged one. This process is repeated until it achieves the desired performance.

The great advantage of FL that made this strategy so attractive to the EI community is that edge nodes exchange and aggregate their local ML models, thereby preserving data privacy, while at the same time avoiding extra computation, and reducing communication overhead when ML model sizes are sufficiently smaller than data sizes [26].

There are different implementations/versions of federated learning trying to overcome possible weak points. The FedCS [39], for example, is an FL protocol that focuses on client selection. In cases of heterogeneous clients, clients with more data, compared to others, will require a longer time to update models unless they have better/higher computational resources. In this protocol, clients notify the server of their resource information. Using this information, the Mobile Edge Computing (MEC) operator determines which clients should be chosen to complete the subsequent steps within a certain deadline.

Abeshu and Chilamkurti [71] present a different approach, where both server and clients are edge nodes, but belong to distinct hierarchies. Then, the so-called worker nodes (clients in FL) train independently and send the weights and biases to the master node (server in FL). The master node, therefore, sends these updates to the other nodes.

The architecture of Fog-embedded Privacy-Preserving Deep Learning (FPPDL) [72] presents an extra layer to the default FL. The data are collected by the end nodes (devices) that forward the transformed data to a nearby fog node. The intermediate nodes are responsible for the client function of the classic FL, computing model gradients based on the data received.

In conventional federated learning, the parameters of the entire Deep Neural Network (DNN) structure are updated at the same time, contributing to a huge communication overhead. The parameters of the shallow layers help the system to learn general features of the content access. On the other hand, a large number of parameters are generated at the deep layers to learn specific features related to specific content features and context information of the end nodes. In Fadlullah and Kato [78], as a consequence, the parameters of the shallow layers could be updated more frequently in contrast with those of the deep layers in an asynchronous fashion.

Doku and Rawat [79] describe a proposal of a network that incorporates federated learning with blockchain, called iFLBC edge. It brings the novelty of designing and employing a Proof of Common Interest (PoCI) mechanism to deal with the scarcity of relevant data, which ensures that the data used to train models in the network are trustworthy. The Federated Learning method is used to gather local updates of potential members of a shard and to generate an averaged global model update that is shared by the members and, then, stored on a blockchain that is unique to each Interest Group. Members of an Interest Group can later download the shared model to provide EI to clients that request it.

#### 4.2.2. Model Partitioning

**Figure 3:**
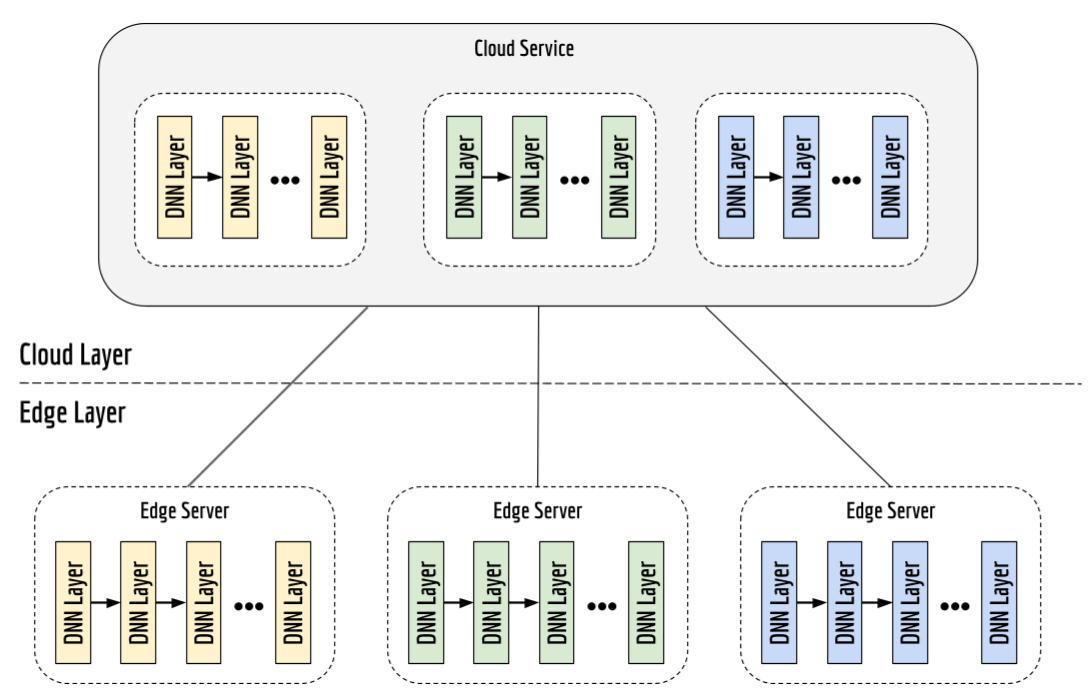
Model Partitioning.

DNN partitioning is the paradigm of distributed machine learning which segments a DNN model into some successive parts and deploys each part on different sites. Figure 3 depicts the elements of Model Partitioning.

Complying with this paradigm, the authors Li, Ota, and Doung [35] use DL in edge computing to process multimedia information. The lower layers of a DNN—the ones closest to the input layer—are offloaded onto edge servers and the others onto the cloud. In DL, the intermediate data are smaller than the initial data. In this way, this technique reduces traffic in the network. In addition, as the initial data undergo some modifications to an intermediate state before being sent to the cloud, the technique can also contribute to the privacy problem.

Metha and Shourey [80] design a new Convolutional Neural Network (CNN) splitting algorithm to efficiently distribute CNN between edge and cloud to reduce bandwidth consumption. Since random layer partitioning may increase bandwidth consumption, several parameters are taken into account to select the optimal splitting layer, such as input image dimensions, bandwidth constraints and task load at the border of the network. The authors show that the best splitting occurs at layers with lower output dimensions than the input images. Because the pre-trained CNN architecture is not modified, there is no loss of accuracy.

Zhang et al. [81] run multiple Kubernetes pods for each edge node. Each pod hosts several docker-containerized Tensorflow jobs that could be categorized as model computation or parameter update jobs for the NN. Model splitting and parallelization occur between nodes. The cloud is responsible for Kubernetes management, with dynamic scaling and maintaining consistency when a node is no longer up but the job is not finished yet.

#### 4.2.3. Right-Sizing

Although the initial layers of the DNN reduce the size of the data, sometimes the intermediate result generated is still large, or the whole process is too slow. Therefore, a method for rapid inference is necessary. Thus, the network must be trained in a different way to generate good results before reaching the end of the network (early-exit), being able to give a result in real-time, without the need to send it to the cloud, thereby reducing the traffic. This method is known as model right-sizing [34], where the DNN model has different exit points and a shorter branch implies a smaller size and thus a shorter runtime. This mechanism focuses on adjusting its size to the limitation of the existing environment.

Regardless the number of devices, the right-sizing method aims at optimizing the use of external resources to accelerate computation. As for DNN right-sizing, the emphasis is placed on tailoring the model size to the constraints of the available environment, which requires advanced training techniques to create a new adjusted model from the original one.

Teerapittayanon et al. [82] propose using Distributed Deep Neural Networks (DDNNs) for fast and localized inference using shallow portions at end devices. It uses DDNNs in the geographical diversity of sensors to improve functions of objects and recognition accuracy and reduce communication costs. Then, it is possible to enhance sensor fusion, system fault tolerance, fast inference and privacy in applications.

The proposals in Li, Zeng, and Chen [34] and Li, Ota, and Dong [68] integrate both mechanisms of DNN partitioning and DNN right-sizing. Suppose the fog nodes are not able to make an accurate inference by themselves. In that case, they will continue to upload the intermediate data of the DNN to the remote cloud servers for further processing. However, if the fog devices or nodes have obtained results that meet their requirements, they can just stop uploading intermediate values and adopt fast inference as the final results.

Zeng et al. [53] present a system called Boomerang, which is designed with two key concepts aimed at meeting the requirement for inference tasks on the manufacturing process. The first key concept is DNN right-sizing, which is employed to accelerate the execution of inference by exiting DNN execution early at a selected intermediate DNN layer. Another key concept is DNN partition, where the DNN computation is partitioned adaptively and distributed to the IoT devices and the edge server according to the available bandwidth.

#### 4.2.4. Edge Pre-Processing

Another mechanism often used to reduce the size of the data in transit through the network is to manipulate it in the edge tier. This method is commonly used in multimedia applications.

In Hossain et al.’s [83] paper, the data captured by end nodes/devices suffer some preliminary processes at the edge and after processed, smaller in size, they are sent to the server (at the cloud). A framework is proposed to classify and reduce environmental noises in conversations through smartphones. Smartphones or recorders send audio to the edge cloudlet via RAN (Radio Access Network) and the it performs the initial processing. Then these data are sent to the core of the network. Cloudlet is a small-scale data center or cloud located at the border of the internet. Its objective is to bring cloud-computing capabilities closer to the consumer. They are typically used for mobile consumers or devices. CNN is adapted and integrated for the Mel-spectrogram. The latter is a good representation of short-time varying sounds, while CNN is good for other types of sounds. Both algorithms integration make big mobile data, integrating an urban environment robust classification system applied to any sound classification.

Hossain [84] presents an example of an audio-visual application. In the example, the edge carries out the speech and video processing such as extraction of pitch and feature extraction. In Liu [37], the author use images from data entry, and they are pre-processed and segmented in the border before being sent to the server where they will pass through a DNN. A similar approach of this mechanism can be found in Wang [29], where the edge is responsible for feature extraction and algorithm selection. The algorithm selected is run in the cloud.

Compressive Sensing (CS) [85] was proposed to sample and compress the signals simultaneously with the sampling rate far lower than the Nyquist sampling rate based on the sparsity. Meanwhile, accurate reconstruction is achievable. Without losing the information, the signal can be sampled with the fewest observations to reduce its dimensions and save the cost of sampling and transmission. CS can be regarded as a cryptosystem when the random measurement matrix is used as a key [86]. However, the measurement matrix used as a key is inconvenient for communication since it has a huge size far larger than the size of plain text; therefore, Chaotic Compressive Sensing (CCS) was proposed to solve the problem of key communication [87]. In other words, a large-size measurement matrix is replaced by a few values.

A formal analysis of results of the employment of edge pre-processing shows how small changes in the prediction accuracy can enable substantial performance improvements. Between the factors that affect the computational cost, there is the number of features extracted from the data. Gómez-Carmona et al. [48], for example, claim that computational effort can be reduced by 80% assuming a decline of the classification accuracy of only 3%. This work utilizes data pre-processing, a three-point median filter to smooth the signal followed by a segmentation process and a feature selection method (Chi2 filtering [88]), which consists of a discriminating process to find essential features that have more weight in the model. It reduces the dimension of the feature matrix by removing the irrelevant features.

For the original sampling provided by the dataset, using only the top three features penalized the results by 3.19%. However, from the baseline parameters (all features and all the signal components) to the final simplified stage (three features and only the most representative component), the time reduction was 81.24% for a laptop, 82.05% for a 540 Raspberry Pi and 92.32% for a Raspberry Zero.

Feature selection powered by swarm search is used as a pre-processing method for improving the accuracy and speed of local Fog data analytics. Fong and Mohammed [63] conduct an experiment testing several feature selection search methods on the Gas Sensor Array Drift dataset, confronting a conventional decision tree algorithm (C4.5) with a data stream mining decision tree algorithm, called Hoeffding Tree (HT), and conclude that fog computing using HT coupled with Harmony feature selection could reach good accuracy, low latency and scalability for this dataset.

Raafat et al. [89] present a new approach for novelty detection in sensor signals based on Levene’s test, which tests the homogeneity of variances of samples taken from the same population and combined with other statistical and autocorrelation features. That is done by extracting seven statistical features into an NN used for event detection (novelty detection), and the computation is carried out at the fog layer. In order to eliminate noise before extracting features, EMD (Empirical Mode Decomposition) is used.

The use of deep learning in edge applications such as augmented reality, real-time video analytic, and others to support decision making is covered by Ali et al. [33]. The authors emphasize very high accuracy levels that were achieved with DNNs and CNNs. The proposed approach performs initial processing of the data close to the source at fog nodes, resulting in a significant reduction in the data that are transferred and stored in the cloud.

Ferdowsi et al. [75] provide a framework for edge computing and analytics architecture applied to vehicular networks. The paper presents a discussion about different machine learning techniques to implement an edge analytics framework in Intelligent Transportation Systems (ITS). This framework allows the transmission of only a summary or basic results extracted from big data to the cloud instead of transmitting the whole amount generated. According to the paper, the framework provides low latency and high reliability.

A similar technique applied to mobile crowd sensing (MCS) was approached by Zhou et al. [74], where raw data such as images or video clips are processed on edge nodes by implementing preliminary filtering, while authentication verification and relevance identification is performed at the border of the network. Unqualified, adulterated and irrelevant information is detected and filtered out, and only the useful sensory information that is preserved is aggregated and uploaded to the sensing platform. In this way, the total amount of data that must be delivered to the sensing platform can be significantly reduced.

Another example is found in the work of Hossain and Muhammad [84] where the authors use a CNN model to recognize emotions through video and speech. In the proposed framework, pre-processing of video and sound is performed at the edge. In addition, the trained parameters of the CNN model are retrieved from the cloud and used at the edge for testing.

#### 4.2.5. Scheduling

A different set of frameworks for addressing EI problems is focused on where to offload the intelligence tasks of the application (edge or cloud). While the mechanism of model partitioning addresses the division of the model between those locations, in the scheduling paradigm, intelligence is implemented at both layers. The application needs to choose at which layer the tasks will more efficiently be offloaded. For example, Ahn et al. [90] propose a two-tier offloading method, in which the optimized decision is made according to the trade-off between low latency and energy saving. Cao et al. [91] split human fall detection between edge devices and servers in the cloud, with front-end IoT devices performing lightweight computation for fall detection, while data are also transmitted to the back-end cloud servers for more intensive computation. The framework proposed by Cao et al. [67] offloads sound manipulation algorithms to both layers, keeping more complicated processing in the cloud.

A framework encompassing storage and offloading techniques for edge computing applications is presented by Hassan et al. [36]. The authors introduce a dynamic offload method according to the capabilities of the edge devices. Based on values of latency, bandwidth, processing and memory, the execution time of the application is predicted to partition it.

The scheduling technique in EI can be employed in many research fields. Wei et al. [41] use scheduling in a satellite network. The satellites are distributed into layers from the edge to the cloud, where the computing capability gradually increases. Computing with low complexity can be performed on the satellite IoT fog node. However, if local computing and storage resources are insufficient, the target detection data may be offloaded to a nearby satellite edge or cloud node. Computing with high complexity and requirements is suitable for completion in the satellite IoT cloud node.

Authors Sun, Liu, and Yue [92] train the NN with large-scale data in the cloud, then train and customize the pre-trained model with small-scale data in the edge and offload tasks to appropriate servers. For mobile devices that do not find an appropriate edge or remote server, the tasks can be accomplished locally at their CPU. For computing tasks with predicted delay to the remote cloud lower than their delay requirement, the computing tasks tend to be routed to the remote cloud, since it is more powerful and can provide the highest accuracy. Therefore, the authors seek to achieve accuracy maximization offloading with latency constraints (AMLC).

Zhou and Chen [93] focus on how to coordinate the edge and cloud to train EI models, with a view to minimizing resource usage. Training data are buffered in a queue before being scheduled to the edge or cloud for processing. They quantify the degree of privacy preservation with the ratio of the total amount that are processed in the cloud to the total amount that arrive in the long term, where a smaller ratio indicates that less data were offloaded to the cloud, and privacy was better preserved. By enforcing the above ratio to remain tolerable, data privacy is promoted.

#### 4.2.6. Cloud Pre-Training

In deep learning applications, where the number of data can be massive, training represents a costly task. Due to the limitations of processing, memory and other considerations, training on the edge is still challenging. Cloud pre-training is another efficient strategy that uses the collaboration between the edge and the cloud to improve data privacy and reduce network traffic.

In applications such as that presented by Lin [94] and Hossain and Muhammad [84] the cloud will perform the computationally expensive training of the models and, once they are trained, the trained parameters are transmitted back to the edge nodes that will be able to execute the inference process.

Moon, Kum, and Lee [42] propose a slightly different approach, where the cloud receives the data and tries to predict an appropriate model for them. The selected one is then sent to the edge. The model manager stores it, allowing the inference module to use it to generate predictions quickly.

To deal with the problem of predicting system disruption in Industry 4.0, Brik et al. [95] propose a framework in which the cloud is responsible for the creation of the model that will be further handled by fog computing to predict a resource’s location in real-time.

Bura et al. [40] present an adaptable and affordable design for a smart parking system. Data collected by cameras and other sensors are processed by advanced deep learning algorithms in edge devices. In this system, the vehicle tracking is performed by a trained Tiny Yolo model run on top of the Nvidia Jetson Tx2. The Tiny Yolo is very light and is, therefore, suitable for devices with limited resources. Moreover, CNN is used in applications of IoT. While comparing the experimental results with other solutions, the system presented good accuracy and a shorter time for inference.

Liu et al. [38] propose pre-training DNNs in the cloud server and sending the weights to the edge server, which operates a deep Q-learning process, presenting a framework with an efficient energy scheduling scheme with deep reinforcement learning.

#### 4.2.7. Edge Only

In the most extreme cases of distributed learning, the computations are all performed in edge devices or nodes, with the cloud server, when used, acting only as host for data storage. Bura et al. [40] and Ke et al. [43] apply this concept to the context of smart parking. Similar methods are proposed by Rachakonda et al. [96] and Hossain [23]. This method tries to bring the computations as close as possible to where the data are collected, reducing the maximum time spent on communications. Privacy and security are also advantages of this method since sensitive and private information will not circulate through the public network.

OpenEI [46] is a framework with a lightweight deep learning Package Manager similar to TensorFlow Lite, optimized to run AI algorithms at the edge, which guarantees low power consumption and low memory footprint. Its Model Selector algorithm looks for the most suitable one for a specific edge platform based on users’ requirements and the capabilities of the hardware platform using the four-element tuple ALEM: "Accuracy, Latency, Energy, Memory footprint". In addition, it supports training the model locally.

Other works, such as those presented by Jiang et al. [73] and Kuo et al. [97], adopt the blockchain infrastructure in their works. This method works like FL, however without a cloud server to distribute the model. Instead, the MEC nodes serve as data storage and model sharing. The models are trained based on distributed deep learning (DDL) [98], meaning that they are trained independently in each node. When they converge, the parameters are chosen by smart contracts; based on an agreement, consensus between two or more parties can be achieved. Blockchain can be seen as a tamper-resistant distributed system to share and store data among a large number of nodes while maintaining the security and privacy of the network [99].

Sanchez et al. [62] introduce a novel algorithm architecture approach to enable real-time low-power CNN processing on edge devices. The core of the proposed approach is utilizing 1D dimensional convolution with an architecture that can truly benefit from the algorithm optimization. The proposed architecture operates near the sensor and avoids storing the streaming data in the main memory. In fact, they avoid unnecessary buffering and create a more latency-friendly and ultimately real-time dataflow architecture.

Lemley, Bazrafkan, and Corcora [100] present different use cases for Edge-AI (Artificial Intelligence in the Edge), such as eye-gaze systems and biometric authentication. A key aspect in this study is that the data generated by these applications require a great level of privacy and security response speed. Therefore, the whole acquisition and processing should occur on the device or on the periphery.

Al-Rakhami et al. [101] propose a lightweight architecture based on Docker [102] to distribute, deploy and manage cloud and edge applications into the clusters, leveraging EI by means of the Regularized Extreme Learning Machine (RELM) algorithm [103], and experimental results suggest a fast training and testing time compared to traditional ELM and Support Vector Machine (SVM) as presented by the Ugulino [104] dataset, also having reported slightly better accuracy.

#### 4.2.8. Model Compression

Model compression is an efficient technique for deploying NN models on edge devices with limited resources by altering the network architecture itself in an attempt to reduce its parameters, and therefore its size.

In this context, He et al. [105] propose AutoML for Model Compression (AMC), using reinforcement learning and achieving model compression in a fully automated way. Zhou et al. [15] perform the compression by removing neurons with low contribution in a network, thus reducing the model size while maintaining its accuracy. Zhang et al. [46] seek deep compression with methods for parameter sharing and pruning, low-rank approximation, and knowledge transfer [106,107]. Deep compression has also achieved remarkable results in SqueezeNet [108].

Bearing the domain of Transportation Cyber-Physical Systems (T-CPS) insight, Zhou et al. [32] introduce a lightweight deep learning model to support MEC applications in the field, aiming to reduce latency and improve context-awareness. It uses Factorization Convolutional (FC) layers, alternating with compression layers, which is named as lightweight CNN-FC by the authors. Experimental results indicate that lightweight CNN-FC significantly decreases the number of unnecessary parameters and model size while maintaining high accuracy compared to conventional CNN.

The Semi-Parallel DNN (SPDNN) [109] combines a number of deep architectures to produce a final model that takes advantage of specialized layers of each architecture while being much smaller than the combined sizes of these networks.

A traditional approach for compressing NN models is called pruning, which seeks to remove redundancies in over-parameterized networks [65], thus reducing the number of parameters without significant impact on the results. Different pruning techniques can be applied either in training [110,111] or during the inference stage [64,112].

Model compression can also be attained with quantization [113] by representing weights and activations of a NN with reduced precision. For instance, Palossi et al. [45] use data quantization to reduce the numerical representation of weights and activations from 32 to 16 bits, as well as modify the receptive field of max-pooling layers from 3 × 3 to 2 × 2, producing the same final results.

The extreme use of quantization leads to Binary NNs (BNNs), where the activations and weights are reduced to binary representations, with massive reductions in resource usage and costs for edge computing. Nonetheless, binarization must be applied very carefully to not prompt drastic performance and scalability issues in complex tasks.

Liu et al. [55] propose assessing the degree of redundancy of each layer before applying binarization since its use on layers with a higher degree of redundancy will ultimately lead to lower performance loss, while layers with a negative degree of redundancy should be kept un-binarized.

Several algorithms fail to counter the degradation caused by binarizing weights and activations completely. To confront this problem, Hybrid-Net [65] applies the Principal Component Analysis (PCA) in a reverse manner: instead of its traditional use as a dimensionality reduction technique, PCA is used here to identify layers that contribute to the most relevant transformations, based on their ability to expand data into higher-dimensional space, where it could be linearly separable. Then the bit precision in the inputs and weights of these significant layers increases. At the same time, the remaining layers are kept entirely binary, producing a successful mixed-precision network topology in the challenge of optimized and highly accurate quantized NNs with binary representations.

**Figure 4:**
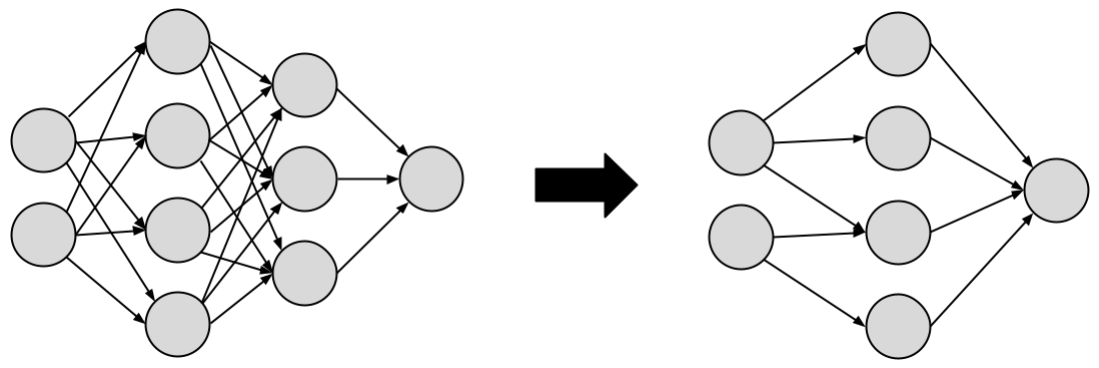
Model compression example.

Lu et al. [49] explore the connection between Binary and Spiking Neural Networks (SNNs) to seek a reduction in computation time and a considerable reduction in model size, relying on competitive accuracies of SNNs in large-scale image recognition datasets, such as ImageNet. The authors apply standard training techniques for non-spiking networks to generate their SNNs with a conversion process and explore design and run-time optimization techniques to reduce inference time for binary and full-precision models. In Figure 4, an example of the technique of model compression is shown.

Table 7 shows the studies grouped by the eight main techniques of edge intelligence.

#### 4.2.9. Other Techniques

Other approaches that differ from the previously discussed techniques have been identified in recent edge intelligence studies.

Gossip Training, for instance, is a technique where each node updates its hosted DNN model locally during the gradient update step and then shares its information with another randomly selected node in the mixing update step. These steps are repeated until all the DNN converge and reach a consensus.

The aim of GoSGD [114] is to address the issue of speeding up the training of convolutional networks. Instead, another gossip-based algorithm—gossiping SGD [115]—is designed to retain the positive features of both synchronous and asynchronous SGD methods. Gossiping SGD replaces the all-reduce joint operation of synchronous training with a gossip aggregation algorithm, achieving asynchrony.

Kamath et al. [69] present a decentralized stochastic gradient descent method to solve large linear regression problems that can be applied in learning/predicting seismic anomalies via real-time imaging. The decentralized reduce operation of the algorithm is based on Gossip Averaging. The method is then applied to a real problem using an edge computing testbed. Results showed it outperformed many alternative methods.

In Da et al.’s [116] approach, parallel transfer learning is used between edge nodes, where aggregation models allow straightforward parallelization to distribute the computations on individual experts. The authors propose a new factorized training strategy and principled aggregation model, named Tr-BCM, for transfer learning to accelerate full transfer Gaussian processes with large-scale source inputs.

Similarly, Hypothesis Transfer Learning (HTL) is used by Valerio et al. [21] with a deep learning pipeline of processing stages across the edge, cloudlet, or fog resources. HTL is a standard machine learning technique used to train models on separate disjoint training sets and then transfer the different parts—instead of data—to reach a unique learning model. In addition to the quick and dynamic adapting models using on-device resources, the technique also bolsters the privacy of user-specific information while keeping it on a local device.

Rincon et al. [117] present an assistant robot based on EI, which incorporates two devices capable of classifying emotional states and physical activities performed by the user. The training uses Keras [118], and after obtaining the H5 model, there is a transformation to TensorFlow lite and, finally, the K model is obtained with a Mobilenet network [119].

Xu et al. [47] use a central fog node as responsible for health data pre-processing with load management and model-ensemble-based prediction with a fully connected NN. In order to achieve load balance, multiple nodes are designed for collaborative analysis.

In Guo et al.’s [50] work, a Non-Intrusive Load Monitoring (NILM) algorithm takes real-time measurements, including power, current, and power factor. It identifies the type of appliance considering a dataset for appliance characteristics. There is an offline training phase and an online application phase. In the training phase, the database for appliance characteristics is established using given data of the measurements and the corresponding type of appliances. When the measurement data are acquired, raw data balancing is performed, then feature scaling is conducted. The result is fed to the machine-learning-based NILM classifiers. The training process can be completed on a computer to handle a large amount of information, but it should be guaranteed that the trained classifiers can be applied in the microcontrollers of smart plugs.

Two distributed machine learning methods based on Deep Reinforcement Learning (DRL) were implemented by Liu et al. [38]: edge DRL and coordinated DRL. The system is distributed over the network. In the first approach, the entire learning system is implemented at the edge layer. In the coordinated strategy, the cloud processes a DNN and its outputs become the input of a Q-Learning method implemented at the border. So, there is a hybrid method with reinforcement learning that is operating on edge devices. These methods were compared with the system completely implemented in the cloud. Coordinated DRL showed less resource consumption and less delay.

Shao et al. [120] propose a Lightweight Residual Network (LRN) architecture and a framework for image de-raining on resource-constrained edge cameras. The LRN can improve the visual quality of images under heavy rain at the expense of marginally compromising the Peak Signal to Noise Ratio (PSNR). To decrease the network complexity, they employ an Inverted Residual Block (IRB) [121] as the basic building block, with significantly low computational cost as it replaces standard convolution with a sequence of pointwise convolutions and depthwise convolutions. A pointwise convolutional layer expands the number of input feature maps, and then a depthwise convolutional layer extracts features, which are linearly combined by the last pointwise convolutional layer. In order to keep space complexity low, each layer uses stride 2 to down-sample the feature maps into a smaller size, then upsample them back using deconvolutional layers.

Kulkarni et al. [122] implement a novel NN architecture to integrate industrial Domain Knowledge (DK) with machine learning in the context of Prognostic Health Management (PHM) solutions for industrial applications. A distributed computational architecture for motion control with edge intelligence and cloud processing is adopted.

Liu and Zhang [123] study how to improve the overall reliability of the time-critical object detection and classification in MEC with the imperfect transmission, where multiple user equipment and edge servers are present, and a certain level of image distortion is tolerated. The authors then formulate an optimization problem to maximize the overall service reliability under latency constraints. The Semidefinite Relaxation (SDR)-based algorithm is designed to find a solution for the association between the user equipment and edge servers and communication and computing resource allocation. The performance of the proposed algorithm is evaluated using the practical object detection methods (SSD [124] and YOLOv2 [125]) in multiple scenarios, showing that the SDR-based algorithm performs similarly to the exhaustive method, at a much lower complexity.

Distributed deep learning applications in an osmotic computing environment are presented by Morshed et al. [70]. The authors focus on biomedical applications, presenting a Holistic Distributed Deep Learning (HDDL) approach to provide the integration of different data and the orchestration of mobile edge, edge and cloud computing in distributed deep learning applications. Some of the main challenges depicted for the development of HDDL reside in how to provide semantic interoperability, privacy, data quality, and volume. The authors propose a high-level HDDL architecture to integrate different systems, especially the biomedical applications that are highlighted in the paper.

### 4.3. RQ3—Frameworks for Edge Intelligence

This section describes the studies that provided answers to the RQ3 of this survey. Table 8 lists the main frameworks currently used in distributed ML applications. The table also correlates each framework with the corresponding EI group of techniques or the main related strategy.

**Figure 5:**
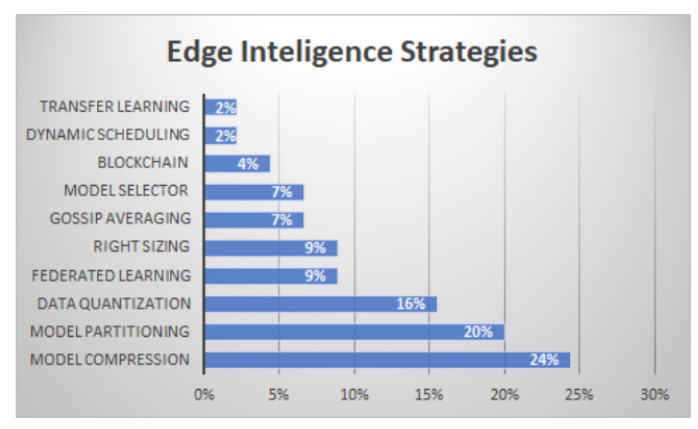
Edge Intelligence strategies.

When correlating the EI strategies with frameworks, it is possible to notice some interesting associations. There are ten of these techniques and strategies, of which only three are present in more than 60% of the papers. They are: (i) Model Compression with 24%, (ii) Model Partitioning with 20%, (iii) Data Quantization with 17%. Federated Learning, Right-Sizing, Gossip Averaging and Model Selector correspond to 9% each. The others have less than 8%. Figure 5 illustrates these ten classes of strategies.

Among these strategies, Model Compression is the most suitable for solving the process of training and testing with the raw data and reducing the dimensionality in real-time. This strategy allows ML algorithms to have faster responses, using lower resources of bandwidth, power and processing. In addition, this technique has proven to be more economical and better at data security once the processing is realized entirely on the edge. In terms of algorithms, the most common is the DNN paradigm of machine learning, which segments models into successive parts (layers). This algorithm allows for the deployment of each part on distinguished sites (model partitioning). DNN also enables compression techniques such as removing nodes or layers, allowing offloading of a whole model in resource-constrained devices.

EI techniques tackle latency problems when part of the entire process is realized on edge devices, decreasing data traffic on the network and, consequently, decreasing the inherent delay in data transmission. Regarding security and privacy issues, it is possible to train and infer on sensitive data partially or fully at the edge, preventing their risky propagation throughout the network, where they are susceptible to attacks.

### 4.4. RQ4—Edge Intelligence Application Domains

**Figure 6:**
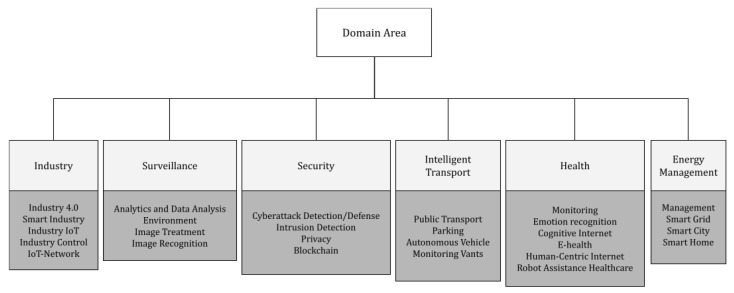
EI application domains.

**Figure 7:**
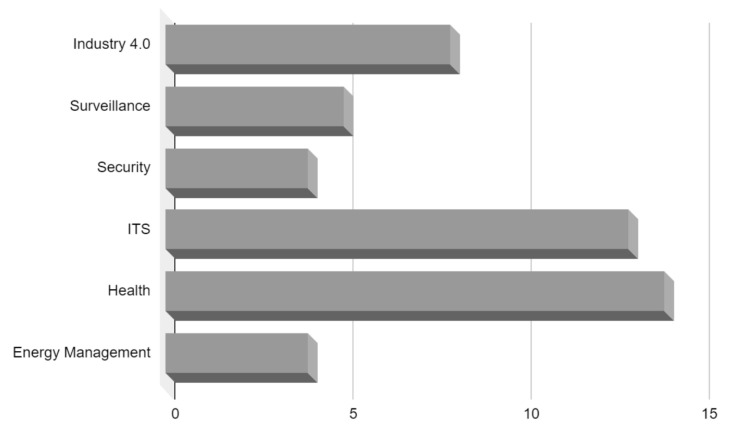
Publications by domain application.

In this section, we present a taxonomy to characterize the application domains where the field of EI has been adopted, providing inputs to answer the RQ4. According to the researched articles, it was possible to group them into six main domains: (i) Industry, (ii) Surveillance, (iii) Security, (iv) Intelligent Transport, (v) Health, and (vi) Energy Management. This does not mean that other domains cannot be created due to new research. Figure 6 illustrates this taxonomy up to a third level. Table 9 shows the works that tackle these domains. Figure 7 summarizes the statistics of the six domains of the publishing by field.

#### 4.4.1. Industry 4.0

Thanks to the development of the Internet of Things (IoT), more precisely the Industrial Internet of Things (IIoT) incorporating Artificial Intelligence (AI) and big data technologies, a new revolution in industry is possible, giving birth to the concept of Industry 4.0, the smart industry. In this new concept, sensors are spread throughout the entire industrial plant, collecting a huge amount of data in real-time. Traditionally, the data are sent to the cloud, where they are processed and analyzed by AI algorithms. The output of the processing is intelligent solutions that can improve manufacturing efficiency and inspection [68], enhance product quality, reduce cost, pollution and risk in industrial production, reducing manual labor and time spent.

IIoT becomes a powerful tool in a big data era, where industrial companies are confronted with market pressures in managing both product quality and manufacturing productivity [53]. According to GE Digital, IIoT is estimated to benefit 46 percent of the global economy [154]. However, with the rise of big data sent to the cloud platforms in IIoT, some problems such as high latency and data privacy are emerging. EI is closer to the user in terms of geographical position and network distance, bypassing the bottleneck of network bandwidth, latency and cost [8]. Moreover, restricted data may be pre-processed before being sent to the cloud or not be sent at all.

#### 4.4.2. Surveillance

Surveillance applications such as seismic imaging [69], air pollution prediction [42], smoke detection [146] and human activity recognition often require systems that can monitor ongoing activities in nearly real-time through object tracking and detection [155], action and activity recognition, event detection, and scene understanding [156,157,158]. Therefore, latency is a critical aspect of this domain, which validates the recent works in this field using edge intelligence as an alternative to the cloud.

#### 4.4.3. Security

The increase in the number and diversity of smart objects has raised substantial concerns about the vulnerability of IoT systems. The new and emerging IoT applications, and even further the concept of the smart city, require novel cybersecurity controls, models, and decisions distributed at the edge of the network to tackle challenges such as attack [71], intrusion [29] and anomaly detection [147], trust management [9], data privacy and others.

The cloud can provide a set of desirable features such as sufficient computing power, memory, energy, etc. However, in a distributed system, some security applications might require a very short response time, and some might produce a large quantity of data (big data) which causes a burden for the network. As a result, cloud computing may not be efficient enough to support these applications [29].

Motivated by the advent of edge computing, a great number of works have been proposed to improve the existing intelligent security applications in this new paradigm of distributed systems. In general, these frameworks can provide better results in comparison to non-distributed ones as the data can be processed at the edge for a shorter response time, helping to reduce the workload for the central server and the delay.

#### 4.4.4. Intelligent Transport System (ITS)

Smart cities are cities that incorporate information and communication technologies (ICT) to improve the quality and performance of urban services such as communication, governance, safety, energy, sustainability and transport.

The Internet of Vehicles (IoV) is an emerging paradigm that is driven by recent advancements in vehicular communications, networking and processing power [148]. This new conjecture enables a plethora of new exciting applications [26,40,43,45,51,52,56,58,75,148,149,150,151] that go beyond the concept already well disseminated of autonomous vehicles as smart parking systems, unmanned aerial vehicles (UAVs), traffic monitoring, intelligent roadways, etc.

However, such as in the other domain fields analyzed in this paper, centralized processing also presents important drawbacks in the ITS subject. Autonomous systems, in general, require real-time response since any delay or latency in transmitting information could be extremely dangerous. Furthermore, monitoring services are based on sound, image and/or video analysis which represents tons of megabytes of data per second that are estimated to increase [159].

Zhang et al. [51] use EI to achieve higher results in QoS and Quality of Experience. It is based on the principle that the infrastructure must provide mechanisms of collaborative sensing and cognitive services to Cognitive Internet of Things applications. As a proof of concept, the authors implemented four applications and services. One of the services is Content Sharing of Vehicular Networks that uses the strategies Heterogeneous Information Network (HIN), Latent Semantic Indexing (LSI) and Collaborative Filtering (CF) to determine the popular content for storage in caches of Roadside Units.

#### 4.4.5. Health

Nowadays, even though we live in a so-called globalized world, insufficient medical resources are still a global problem according to the World Health Organization (WHO) [160]. Patients in the countryside or developing countries have difficulty obtaining high-quality and timely healthcare because of geographical barriers.

The recent effort in integrating IoT into the healthcare scenario has been a great catalyst to the development of novel smart care applications or the enhancement of past ones, which may include telemedicine, tele-consultancy, pervasive health monitoring applications, human activity [101] and/or emotion recognition [117], and others.

Recently, the topic of data privacy has gained prominence in the industry, news and courts. When referring to health data, it is no different. On the contrary, it requires even more concern, as we are talking about extremely sensitive data. In response to the demand for privacy, trust and control over the data, executing machine learning tasks at the edge of the system has the potential to make the healthcare services more human-centric [48].

Besides the easy-to-notice benefits to latency-sensitive and aforementioned data-sensitive tasks, edge AI can also allow healthcare to be less costly, more efficient and incredibly beneficial in the field of cognitive assistants for the elderly, people living alone, or the people geographically segregated. EI is an interesting tool for the democratization of healthcare services.

#### 4.4.6. Energy Management

In recent years, energy management systems (smart grid [51], smart buildings [24], smart plugs [50] and so on) have received huge research and industrial attention with the explosive development of smart cities [38]. As one of the most critical urban services, electric power systems play a vital role in supporting our society and economy. Therefore, it is incumbent on smart cities to propose solutions to energy management by ubiquitous monitoring and reliable communications.

IoT manufacturers and application developers are devoting themselves to developing frameworks to store sensed data in edge nodes and perform data processing via analytics at the edge of the network.

Within the wide scope of this field, the smart grid is a proposition to improve the performance of the standard electric network and manage the various distributed energy resources by continuously monitoring the dynamics of electricity consumption through a considerable number of sensors scattered over the network.

In a narrower scope of smart homes, there is a typical IoT device, smart plugs (or smart outlets) [161]. A smart plug can monitor the power usage of the appliance plugged into it in real-time, transmit it as well as other measurements to the processing server (edge or cloud), and receive remote commands or settings to control the operation of the connected device, turning conventionally passive devices into “smart” ones [50].

## 5. Open Issues and Future Directions

Although the EI research field has been gaining traction and becoming a global trend, some issues are still open, without a concrete solution at this stage.

Security and privacy are very important topics that concern both the industry and academia. How to store data and make it available in a safe and privacy-aware way for training and inference [27]? As presented throughout the text, many techniques, such as model compression, face the burden of managing the tradeoff between low latency and/or light solution and inference accuracy [26].

The CAP (Consistency, Availability e Partition tolerance) Theorem [162] states that it is impossible for a distributed data store to simultaneously provide more than two out of the following three guarantees: consistency, availability and partition tolerance. This concept can be translated pretty well to the paradigm of EI, once it involves distributed data storage, transport and processing [71]. Moreover, Zhang et al. [51] mention the difficulty of achieving data consistency on edge devices in an efficient and distributed approach.

Another point that requires the attention of the research community is the adaptability of statically trained models [71]. Since edge devices are known for not having a high power of processing and/or storage, some strategies take the training step to be run in a controlled and powerful environment. Subsequently, the model is offloaded to the nodes. However, this translation has still not been perfected.

New researches increasingly refer to the growing demand for research related to the heterogeneous environment present at the edge, with the need for ubiquitous computing, communication, and caching resources. According to Zhang et al. [163] and also Ke et al. [43], the strength of 5G technology is likely to be an essential enabler for the IoT intelligence environment. This technology, by offering intelligent cloud services and close to the production environment with low latency and lower cost, should allow the evolution of many other lines of research to support edge intelligence, as well as for the improvement in terms of network security and preventing vulnerable transactions from malicious nodes.

## 6. Conclusions

Edge intelligence refers to the ability to bring the execution of machine learning tasks from the cloud closer to the IoT devices, either partially or entirely. Some synonyms of this concept found in the literature include distributed learning, edge/fog learning, distributed intelligence, edge/fog intelligence and mobile intelligence. In this work, we presented a survey on distributed edge intelligence, debating its challenges: (i) limited resources; (ii) ensuring energy efficiency of edge devices; (iii) communication efficiency (iv) ensuring data privacy and security; (v) handling edge device failure; and (vi) heterogeneity and quality of data.

We established our research methodology in compliance with a thorough Systematic Literature Review protocol to attain a comprehensive, impartial and auditable process of review. By analyzing the results of our literature review, we could identify some promising strategies bringing ML/DL to edge computing, although prevailing studies are still flourishing. So far, we also found a slightly predominant tendency to use CNN and DNN in edge intelligence, but without prejudice to other approaches.

In this survey, we understand that the distributed ML/DL in edge computing is an emerging new area, which opens up many research opportunities to deal with the existing challenges of distributed tasks on restricted devices. In terms of the challenges, we point out the need to offer (i) running of ML/DL on devices with limited resources; (ii) ensuring energy efficiency without compromising the accuracy; (iii) communication efficiency; (v) ensuring data privacy and security; (vi) handling failure in edge devices; (viii) heterogeneity and low quality of data.

Finally, we present in this paper not only a comprehensive overview of what has been investigated and developed for Edge Intelligence but also provide means to direct future researches in this context. We aim to contribute to the more effective development of intelligence at the edge with these analyses.

## Figures and Tables

**Table 1:** Comparison of existing surveys.

	Scope		
Paper	Challenges	Group of Techniques	Different Application Domains
Al-Rakhami et al. [13]	0/6	2/8	1/6
Wang et al. [14]	1/6	4/8	4/6
Verbraeken et al. [16]	1/6	0/8	0/6
Zhou et al. [15]	2/6	4/8	0/6
Dianlei Xu et al. [10]	6/6	3/8	0/6
Our work	6/6	8/8	6/6

**Table 2:** Research Questions (RQs).

	Research Questions (RQs)	Goals
RQ1	What are the main challenges and open issues in the distributed learning field?	To obtain an understanding of the main challenges and open issues in the distributed learning field.
RQ2	What are the techniques and strategies currently used in distributed learning?	To characterize techniques and strategies used in distributed learning.
RQ3	What are the frameworks currently used in distributed learning?	To characterize frameworks used in distributed learning.
RQ4	What are the different application domains of edge intelligence?	To characterize the different application domains of edge intelligence.

**Table 3:** Criteria adopted to include papers in the study.

Inclusion Criteria	
IC1	The study presents or discusses opportunities or challenges to run ML at the edge
IC2	The study presents or discusses applications of ML at the edge
IC3	The study presents or discusses techniques, strategies and/or frameworks that enable ML to run at the edge of the network

**Table 4:** Criteria adopted to exclude papers from the study.

Exclusion Criteria	
EC1	The study is not related to Edge/Fog Computing
EC2	The study is not related to distributed ML in Edge/Fog Computing
EC3	The study is a previous version of a more complete study about the same research
EC4	The study was not approved according to the relevance criteria

**Table 5:** Challenges in distributed machine learning in edge computing.

Challenges	
CH1	Running ML/DL on devices with limited resources
CH2	Ensuring energy efficiency without compromising the accuracy
CH3	Communication efficiency
CH4	Ensuring data privacy and security
CH5	Handling failure in edge devices
CH6	Heterogeneity and low quality of data

**Table 6:** References to the challenges of Edge Intelligence.

	References	Works That Tackle the Challenges
CH1	[10,15,22,23,24,25,26,27,28,29,30,31]	[14,28,31,32,33,34,35,36,37,38,39,40,41,42,43,44,45,46,47,48,49,50,51,52,53,54,55,56,57,58,59,60,61,62,63,64,65,66]
CH2	[10,15,23,26,28,30,41]	[8,24,28,31,33,36,37,38,49,52,56,59,61,65,66,67]
CH3	[10,24,28,29,46,66]	[16,21,23,33,34,35,36,39,43,46,56,68,69]
CH4	[10,24,27,36,44,70]	[8,9,10,24,44,51,71,72,73]
CH5	[10,27]	–
CH6	[10,24,44,74,75]	[10,38,70]

**Table 7:** References considering techniques and/or strategies of edge intelligence implementations.

Techniques	Works
Federated Learning	[39,71,72]
Model Partitioning	[34,35,38,68]
Model Right-sizing	[34,68,82]
Edge Pre-Processing	[29,83,84]
Scheduling	[36,41,67,90]
Cloud Pre-Training	[42,84,94]
Edge Only	[23,38,40,43,73,74,96,97]
Model Compression	[15,45,46,55,64,65,91,105,108,109,110,111,112,113],

**Table 8:** EI frameworks.

Framework	Groups of Techniques or Strategies	Comments
Neurosurgeon [126]	Model Partitioning	Lightweight scheduler to automatically partition DNN computation between edge devices and cloud at the granularity of NN layers
JointDNN [127]	Model Partitioning	JointDNN provides an energy- and performance-efficient method of querying some layers on the mobile device and some layers on the cloud server.
H. Li et al. [35]	Model Partitioning	They divide the NN layers and deploy the part with the lower ones (closer to the input) into edge servers and the part with higher layers (closer to the output) into the cloud for offloading processing. They also propose an offline and an online algorithm that schedules tasks in Edge servers.
Musical chair [128]	Model Partitioning	Musical Chair aims at alleviating the compute cost and overcoming the resource barrier by distributing their computation: data parallelism and model parallelism.
AAIoT [129]	Model Partitioning	Accurate segmenting NNs under multi-layer IoT architectures
MobileNet [46]	Model Compression Model Selector	Presented by Google Inc., the two hyperparameters introduced allow the model builder to choose the right sized model for the specific application.
Squeezenet	Model Compression	It is a reduced DNN that achieves AlexNet-level accuracy with 50 times fewer parameters
Tiny-YOLO	Model Compression	Tiny Yolo is a very lite NN and is hence suitable for running on edge devices. It has an accuracy that is comparable to the standard AlexNet for small class numbers but is much faster.
BranchyNet	Right sizing	Open source DNN training framework that supports the early-exit mechanism.
TeamNet [130]	Model Compression Transfer Learning	TeamNet trains shallower models using the similar but downsized architecture of a given SOTA (state of the art) deep model. The master node compares its uncertainty with the worker’s and selects the one with the least uncertainty as to the final result.
OpenEI [46]	Model Compression Data Quantization Model Selector	The algorithms are optimized by compressing the size of the model, quantizing the weight. The model selector will choose the most suitable model based on the developer’s requirement (the default is accuracy) and the current computing resource.
TensorFlow Lite [131]	Data Quantization	TensorFlow’s lightweight solution, which is designed for mobile and edge devices. It leverages many optimization techniques, including quantized kernels, to reduce the latency.
QNNPACK (Quantized Neural Networks PACKage) [132]	Data Quantization	Developed by Facebook, is a mobile-optimized library for high-performance NN inference. It provides an implementation of common NN operators on quantized 8-bit tensors.
ProtoNN [133]	Model Compression	Inspired by k-Nearest Neighbor (KNN) and could be deployed on the edges with limited storage and computational power.
EMI-RNN [134]	Right Sizing	It requires 72 times less computation than standard Long Short term Memory Networks (LSTM) and improves its accuracy by 1%.
CoreML [135]	Model Compression Data Quantization	Published by Apple, it is a deep learning package optimized for on-device performance to minimize memory footprint and power consumption. Users are allowed to integrate the trained machine learning model into Apple products, such as Siri, Camera, and QuickType.
DroNet [37]	Model Compression Data Quantization	The DroNet topology was inspired by residual networks and was reduced in size to minimize the bare image processing time (inference). The numerical representation of weights and activations reduces from the native one, 32-bit floating-point (Float32), down to a 16-bit fixed point one (Fixed16).
Stratum [136]	Model Selector Dynamic Scheduling	Stratum can select the best model by evaluating a series of user-built models. A resource monitoring framework within Stratum keeps track of resource utilization and is responsible for triggering actions to elastically scale resources and migrate tasks, as needed, to meet the ML workflow’s Quality of Services (QoS). ML modules can be placed on the edge of the Cloud layer, depending on user requirements and capacity analysis.
Efficient distributed deep learning (EDDL) [57]	Model Compression Model Partitioning Right-Sizing	A systematic and structured scheme based on balanced incomplete block design (BIBD) used in situations where the dataflows in DNNs are sparse. Vertical and horizontal model partition and grouped convolution techniques are used to reduce computation and memory. To speed up the inference, BranchyNet is utilized.
In-Edge AI [5]	Federated Learning	Utilizes the collaboration among devices and edge nodes to exchange the learning parameters for better training and inference of the models.
Edgence [137]	Blockchain	Edgence (EDGe + intelligENCE) is proposed to serve as a blockchain-enabled edge-computing platform to intelligently manage massive decentralized applications in IoT use cases.
FederatedAveraging (FedAvg) [77]	Federated Learning	Combines local stochastic gradient descent (SGD) on each client with a server that performs model averaging.
SSGD [76]	Federated Learning	System that enables multiple parties to jointly learn an accurate neural network model for a given objective without sharing their input datasets.
BlockFL [138]	Blockchain Federated Learning	Mobile devices’ local model updates are exchanged and verified by leveraging blockchain.
Edgent [6]	Model Partitioning Right-Sizing	Adaptively partitions DNN computation between the device and edge, in order to leverage hybrid computation resources in proximity for real-time DNN inference. DNN right-sizing accelerates DNN inference through the early exit at a proper intermediate DNN layer to further reduce the computation latency.
PipeDream [139]	Model Partitioning	PipeDream keeps all available GPUs productive by systematically partitioning DNN layers among them to balance work and minimize communication.
GoSGD [114]	Gossip Averaging	Method to share information between different threads based on gossip algorithms and showing good consensus convergence properties.
Gossiping SGD [140]	Gossip Averaging	Asynchronous method that replaces the all-reduce collective operation of synchronous training with a gossip aggregation algorithm.
GossipGraD [141]	Gossip Averaging	Asynchronous communication of gradients for further reducing the communication cost.
INCEPTIONN [142]	Data Quantization	Lossy-compression algorithm for floating-point gradients. The framework reduces the communication time by 70.9 80.7% and offers 2.2 3.1× speedup over the conventional training system while achieving the same level of accuracy.
Minerva [143]	Data Quantization Model compression	Quantization analysis minimizes bit widths without exceeding a strict prediction error bound. Compared to a 16-bit fixed-point baseline, Minerva reduces power consumption by 1.5×. Minerva identifies operands that are close to zero and removes them from the prediction computation such that model accuracy is not affected. Selective pruning further reduces power consumption by 2.0× on top of bit width quantization.
AdaDeep [144]	Model Compression	Automatically selects a combination of compression techniques for a given DNN that will lead to an optimal balance between user-specified performance goals and resource constraints. AdaDeep enables up to 9.8× latency reduction, 4.3× energy efficiency improvement, and 38× storage reduction in DNNs while incurring negligible accuracy loss.
JALAD [145]	Data Quantization Model Partitioning	Data compression by jointly considering compression rate and model accuracy. A latency-aware deep decoupling strategy to minimize the overall execution latency is employed. Decouples a deep NN to run a part of it at edge devices and the other part inside the conventional cloud.

**Table 9:** Application domains and corresponding works.

Domains	Works That Approach the Theme
Industry (8)	[8,31,51,53,68,92,95,122]
Surveillance (5)	[42,69,81,120,146]
Security (4)	[9,29,71,147]
Intelligent Transport Systems (ITS) (13)	[26,40,43,45,51,52,56,58,75,148,149,150,151],
Health (14)	[13,25,28,47,48,51,67,70,84,91,101,117,152,153]
Energy Management (4)	[38,50,51,94]

## Data Availability

The datasets generated during and/or analyzed during the current study are available from the corresponding author on reasonable request.

## Abbreviations

The following abbreviations are used in this manuscript:

AI	Artificial Intelligence
AMC	AutoML for Model Compression
CF	Collaborative Filtering
CH	Challenge
CNN	Convolutional NN
CS	Compressive Sensing
CCS	Chaotic Compressive Sensing
DK	Domain Knowledge
DL	Deep Learning
DNN	Deep NN
DDNN	Distributed DNN
DRL	Deep Reinforcement Learning
DSSGD	Distributed Selective SGD
EC	Exclusion Criteria
Edge-AI	Artificial Intelligence in the Edge
EI	Edge Intelligence
ELM	Extreme Learning Machine
FC	Factorization Convolutional [layers]
FL	Federated Learning
FPPDL	Fog-embedded Privacy-Preserving Deep Learning
GoSGD	Gossip Stochastic Gradient Descent
HDDL	Holistic Distributed Deep Learning
HIN	Heterogeneous Information Network
HT	Hoeffding Tree
HTL	Hypothesis Transfer Learning
IC	Inclusion Criteria
IRB	Inverted Residual Block
IIoT	Industrial Internet of Things
ITS	Intelligent Transportation Systems
LRN	Lightweight Residual Network
LSI	Latent Semantic Indexing
MEC	Mobile Edge Computing
MEMS	Micro-Electro-Mechanical Systems
ML	Machine Learning
NILM	Non-Intrusive Load Monitoring
NN	Neural Network
PHM	Prognostic Health Management
PoCI	Proof of Common Interest
PSNR	Peak Signal to Noise Ratio
QC	Quality Criteria
QoS	Quality of Service
RAN	Radio Access Network
RELM	Regularized ELM
RQ	Research Question
SDR	Semidefinite Relaxation
SGD	Stochastic Gradient Descent
SLR	Systematic Literature Review
SNN	Spiking NN
SPDNN	Semi-Parallel DNN
SVM	Support Vector Machine
T-CPS	Transportation Cyber-Physical Systems
WHO	World Health Organization
